# Crystal Structure of Penicillin-Binding Protein 3 (PBP3) from *Escherichia coli*

**DOI:** 10.1371/journal.pone.0098042

**Published:** 2014-05-29

**Authors:** Eric Sauvage, Adeline Derouaux, Claudine Fraipont, Marine Joris, Raphaël Herman, Mathieu Rocaboy, Marie Schloesser, Jacques Dumas, Frédéric Kerff, Martine Nguyen-Distèche, Paulette Charlier

**Affiliations:** 1 Centre d’Ingénierie des Protéines, Université de Liège, Institut de Physique B5a et Institut de Chimie B6a, Sart Tilman, Liège, Belgium; 2 Sanofi R&D, protein production, 13 quai Jules Guesde, 94403 Vitry sur Seine, France; Centre National de la Recherche Scientifique, Aix-Marseille Université, France

## Abstract

In *Escherichia coli*, penicillin-binding protein 3 (PBP3), also known as FtsI, is a central component of the divisome, catalyzing cross-linking of the cell wall peptidoglycan during cell division. PBP3 is mainly periplasmic, with a 23 residues cytoplasmic tail and a single transmembrane helix. We have solved the crystal structure of a soluble form of PBP3 (PBP3_57–577_) at 2.5 Å revealing the two modules of high molecular weight class B PBPs, a carboxy terminal module exhibiting transpeptidase activity and an amino terminal module of unknown function. To gain additional insight, the PBP3 Val88-Ser165 subdomain (PBP3_88–165_), for which the electron density is poorly defined in the PBP3 crystal, was produced and its structure solved by SAD phasing at 2.1 Å. The structure shows a three dimensional domain swapping with a β-strand of one molecule inserted between two strands of the paired molecule, suggesting a possible role in PBP3_57–577_ dimerization.

## Introduction

The penicillin-binding proteins (PBPs) synthesize and remodel the cell wall peptidoglycan, a major component of the bacterial cell wall that gives the cell its shape and rigidity [1]–[4]. They are found in all bacteria and represent major targets in anti-biotherapy, especially for the widely used β-lactam antibiotics. Penicillin-binding proteins belong to the family of acyl-serine transferases and are traditionally separated into high-molecular-weight (HMW) PBPs and low-molecular-weight (LMW) PBPs based on molecular weight and sequence homology [4]–[6]. The former enzymes act as transpeptidases *in vivo* and are involved in peptidoglycan synthesis while the latter are carboxypeptidases and endopeptidases [7] thought to remodel peptidoglycan during the bacterial life cycle but details of their *in vivo* activities are not well established. The HMW-PBPs group can be subdivided into classes A and B, the LMW group into classes A, B and C. Class B HMW-PBPs can be further divided into subclasses and *Escherichia coli* PBP3 is paradigmatic of subclass B3 that groups class B PBPs from Gram negative bacteria involved in cell division. Some PBPs from Gram positive bacteria involved in spore peptidoglycan synthesis also belong to subclass B3, e.g. SpoVD from *Bacillus subtilis*.

During cell division, the peptidoglycan is synthesized by the periplasmic part of a macromolecular complex called the divisome, made up of at least 20 proteins in *Escherichia coli* [8]. Cell division is initiated by the polymerization of tubulin homolog FtsZ into a contractile ring at midcell [9] and the other division proteins are recruited sequentially to the septal ring. FtsZ first associates with FtsA, ZapA and ZipA that stabilize the FtsZ filaments and tether them to the cytoplasmic membrane. The divisome then matures with a long delay between formation of the Z ring and recruitment of the proteins downstream of FtsK[10]. These proteins involved in septal peptidoglycan synthesis are now thought to be recruited as subcomplexes, at least FtsQ/L/B [11] and FtsW/PBP3 [12]. FtsN, which contains a SPOR peptidoglycan binding domain, is the last essential division protein that localizes at the septum [13]. Finally, various proteins not essential for septal peptidoglycan synthesis associate with the divisome: the Tol/Pal complex involved in the invagination of the outer membrane [14], and the peptidoglycan hydrolase AmiC (and its activator NlpD) that plays an essential role in the separation of daughter cells [15]. Recently, the outer membrane protein LpoB was also shown to associate with the divisome and to regulate peptidoglycan synthesis by interacting with the glycan chain polymerase/transpeptidase PBP1b [16].

In *E. coli*, PBP3 (FtsI) is an essential protein of the divisome, catalyzing peptide cross-bridges between the glycan chains of the peptidoglycan. PBP3 is involved in many interactions within the divisome. It interacts directly with PBP1b which localizes at the division site during septation in a PBP3 dependent fashion [17]. PBP3 works in concert with PBP1b to incorporate the nascent glycan chain into the existing peptidoglycan [4]. The N-terminal 56 residues of PBP3 (containing a cytoplasmic peptide, the transmembrane segment and a short periplasmic peptide) interact with PBP1b in a two-hybrid assay. However, other interacting sites should be present in the periplasmic part of PBP1b and PBP3 [17]. PBP3 also interacts directly with FtsW and with FtsN, which itself interacts with PBP1b and stimulates its activity [18]. These proteins are able to form a discrete complex independently of the other cell division proteins [12]. Interaction of PBP3 with FtsA, FtsK, FtsQ [19] or FtsL [20] were also reported but the structural details and the sites of interaction between PBP3 and the proteins of the divisome remain to be elucidated.

The *ftsI* gene encodes a 588 residues protein but proteolytic cleavage removes 11 amino acids at the C-terminal part of the protein [21]. We have solved the crystal structure of the periplasmic domain of PBP3 (residues 57–577) at 2.5 Å. We have also produced, purified, crystallized and solved the structure of the Val88-Ser165 subdomain (PBP3_88–165_), a potential protein-protein interaction domain, which was poorly defined in the electron density map of PBP3_57–577_.

## Materials and Methods

### Bacterial Strains, Oligonucleotides and Media

Bacterial strains were *E. coli* Top 10F’ for cloning (Invitrogen, USA) and *E. coli* C41(DE3) for expression [22]. Oligonucleotides were from Eurogentec. The rich media used were 2XYT (bacto-tryptone 16 g, yeast extract 10 g, NaCl 5 g, water 1 l, pH 7.0.) or Luria-Bertani (LB) medium supplemented with ampicillin (100 µg ml^−1^), chloramphenicol (20 or 30 µg ml^−1^), kanamycin (50 µg ml^−1)^ and IPTG (0.5 mM) when appropriate.

### PBP3_57–577_: Production, Purification, Crystallization, Data Collection and Structure Refinement

Plasmids used were pDML232 for the PBP3 and pDML237 for SecB [23]. Fermentation of *E. coli* strain W3110M was performed in RFB MIL11/03 at 37°C to overexpress the PBP3(57–577), allowing to obtain soluble protein expression at 210 mg/l of culture.

50 g of wet bacterial pellet corresponding to 1 litre of culture were suspended into 150 ml of 100 mM Tris (pH 8), 0.1 mM PMSF buffer, under magnetic stirring in an ice batch for 30 minutes. Mechanical lysis of bacteria was performed with a Rannie at 650 bars and cooling. Cell lysate was centrifuged on a JOUAN SR 20.22 at 42 000 g for 1 hour at 4°C. We then centrifuged the supernatant on a Beckman XL90 at 100 000 g for 30 minutes at 4°C, in order to clarify the solution.

The clarified supernatant was loaded on a S-Sepharose Fast Flow column (XK50/30) equilibrated with buffer A (100 mM Tris (pH 8), 10% glycerol, 10% ethylene glycol) at 608 ml/h. Elution was performed with a linear gradient from 100% of buffer A to 40% of buffer B (buffer A + 1 M Nacl). PBP3 was eluted at about 20% of buffer B. Eluate was diluted 1/3 in buffer A and loaded on an S-Sepharose Hiload (XK16/10) column equilibrated in buffer A at 588 ml/h. Elution was performed with buffer C (100 mM Tris (pH 8), 10% glycerol, 10% ethylene glycol, 0.5 M NaCl). The eluate collected in one column volume was then purified on a Superdex 200 (XK50/60) column at 294 ml/h to obtain a highly purified and homogenous protein. 120 mg of purified protein were obtained at 1.6 mg/ml (UV measurement). N-terminal sequence was checked and confirmed. Circular dichroism analyses showed that the protein has a stable three-dimensional structure with 30% of alpha-helix. The FRET (resonance energy transfer) measurements showed a rotational coefficient of 38 ns which demonstrated the monodisperse status of the population with an apparent molecular weight of 53 KDa.

Crystals of PBP3_57–577_ were grown at 20°C by hanging drop vapor diffusion. Crystals were obtained by mixing 5 µl of a 18 mg/ml protein solution (also containing 0.5 M NaCl and 20 mM Tris, pH 8), 4 µl of well solution (2.5 M ammonium sulfate and 0.1 M N-cyclohexyl-3-aminopropanesulfonic acid (CAPS), pH 10), and 1 µl of 0.1 M NaCl solution. Crystals appeared after several months and the apparent very narrow range of crystallization conditions resulted in only some very small badly diffracting crystals and one crystal diffracting at 2.5 Å. Diffraction data were measured on Beamline ID29 at the European Synchrotron Radiation Facility (ESRF, Grenoble, France) and processed using Mosflm [24] and SCALA from the CCP4 program suite. [25] The structure of PBP3 was solved by molecular replacement with the program PHASER [26] using the structure of PBP2 from *Neisseria gonorrhoeae* (PDB id: 3equ) as the initial search model. Refinement was carried out using REFMAC5, [27] TLS, [28] and Coot. [29]. The final refinement statistics are given in Table 1.

**Table 1:** Data collection and refinement statistics.

Crystal	PBP3_57–577_	PBP3_88–165_SeMet derivative	PBP3_88–165_
PDB code	4BJP		4BJQ
Data Collection:			
Space group	P6_1_22	P1	P1
Cell Dimensions			
a, b, c (Å)	119.0, 119.0, 139.2	55.8, 55.8, 81.5	56.0, 56.0, 82.3
α, β, γ (°)	90, 90, 120	75.8, 89.4, 65.3	76.2, 89.1, 66.0
Resolution range (Å)^a^	82.8−2.5 (2.64−2.5)	49−2.7 (2.85−2.70)	38.9−2.10 (2.21−2.10)
No. of unique reflections	20753	45763	45669
Rmerge (%)^a^	16.6 (54.3)	11.7 (47.8)	8.0 (50.8)
<I>/<σI>^a^	13.5 (4.9)	8.7 (2.6)	10.2 (2.5)
Completeness (%)^a^	99.8 (98.8)	95.4 (93.8)	88.5 (95.4)
Redundancy^a^	14.0 (10.4)	2.6 (2.6)	3.7 (3.8)
Refinement:			
Resolution range (Å)	59.5−2.5		35.7−2.1
No. of non hydrogen atoms	3409		5467
Number of water molecules	135		533
R cryst (%)	19.9		20.8
R free (%)	24.5		26.2
RMS deviations from ideal Stereochemistry			
Bond lengths (Å)	0.012		0.010
Bond angles (°)	1.41		1.19
Mean B factor (all atoms) (Å^2^)	34.1		31.9
Ramachandran plot^b^			
Favoured region (%)	98.5		99.7
Allowed regions (%)	1.5		0.3
Outlier regions (%)	0		0

a Statistics for the highest resolution shell are given in parentheses.

b Using program rampage [59].

### PBP3_88–165_: Production, Purification, Crystallization, Data Collection and Structure Refinement

The *ftsI* fragment encoding PBP3_88–165_ was amplified by PCR using plasmid pMVRI [17] as template and oligonucleotides 5′-GGACCCGGGGTAAAAGCGATTTGGGCTGACCC-3′ and 5′-GCCGGATCCTTAAGAC TCTTCACGCAGATGAATCCC-3′ as primers (*XmaI* and *BamHI* are underlined). The PCR fragment was cloned into the pJet1.2/blunt cloning vector (Fermentas), sequenced, digested with *XmaI* and *BamHI* and inserted into the same sites of plasmid pET-52b(+). The resulting plasmid pDML2042 codes for the PBP3_88–165_ with an N-terminal strep-tag. The strep-tag- PBP3_88–165_ was isolated from *E. coli* C41(DE3) harbouring pDML2042 grown at 37°C in 2XYT medium in the presence of 0.5 mM IPTG for 3 h. The harvested cells were suspended in 40 ml of 100 mM Tris-HCl pH 8.0, 150 mM NaCl, 1 mM EDTA containing a protease inhibitor cocktail (Roche) (buffer C), broken 5 times into a high-pressure homogenizer (Emulsiflex-C3 Avestin Inc.) and centrifuged at 25000 g for 40 min. The supernatant was filtered (0.45 µ) and applied to a 5 ml *Strep*-Tactin IBA column. After 5 washes with buffer C, the strep-tag-PBP3_88–165_ was eluted in 100 mM Tris-HCl pH 8.0, 150 mM NaCl, 1 mM EDTA, 2.5 mM desthiobiotin. The fractions of interest (5 ml) were dialyzed against 2 L of buffer C with a 3,500 Dalton cut off membrane and analyzed by SDS-18% PAGE. About 6 mg of PBP3_88–165_ per liter of culture were produced and purified to 90% purity. The strep-tag was removed from PBP3_88–165_ before crystallization.

Crystals of PBP3_88–165_ were grown at 20°C by hanging drop vapor diffusion. Crystals were obtained by mixing 4 µl of a 7.5 mg/ml protein solution containing 0.15 M NaCl and 0.1 M Tris, pH 8 1 mM EDTA, and 1 µl of well solution (2 M ammonium sulfate and 0.1 M citrate, pH 3.5). The structure of PBP3_88–165_ was solved by single anomalous diffraction using a selenomethionine substituted SePBP3_88–165_ crystal. Selenomethionine substituted SePBP3_88–165_ was expressed by using minimal medium supplemented with selenomethionine and purified and crystallized as PBP3_88–165_. Diffraction data for the SePBP3_88–165_ crystals were measured on Beamline PROXIMA 1 at SOLEIL (Paris, France). Data were processed using XDS [30] and initial structure determination of SePBP3_88–165_ was determined with the help of SHELXC/D/E [31], Parrott [32] and Buccaneer [33].

Refinement was carried out on native PBP3_88–165_ using REFMAC5, [27] TLS, [28] and Coot. [29]. Diffraction data for the native PBP3_88–165_ were measured on Beamline BM30A at the European Synchrotron Radiation Facility (ESRF, Grenoble, France) and processed using Mosflm [24] and SCALA from the CCP4 program suite. [25] Data and final refinement statistics are given in Table 1.

### Western Blotting

Western blotting was carried out as described [12]. PBP3, PBP1b and FtsN were revealed with respective polyclonal antibodies and FtsW was probed with monoclonal anti-HA-Peroxydase (HighAffinity (3F10) Roche).

### Light Scattering (DLS and SLS)

Dynamic and static light scattering data were recorded on a DynaPro NanoStar instrument (Wyatt Technology Corporation) operated in batch mode at 20°C and fitted with a laser beam emitting at 658 nm with power auto-attenuation. Scattering angles were 90° for both DLS (avalanche photodiode) and SLS (silicon PIN photodetector). Measurements were performed under buffer conditions and concentration used for crystallogenesis. Samples were filtered on Whatman Anotop 10 inorganic membrane (0.02 µm cut off) and loaded into a 10 µl quartz microcuvette. Data were averaged from 20 acquisitions of the scattered light intensity during 5 s, with a sum of squares error value below 100. Scattering data were analyzed using DYNAMICS v. 7.1.1.3 software (Wyatt Technology Corp.) that includes the DYNALS module for distribution analysis in photon correlation spectroscopy. A globular protein model was used for mass estimation in DLS and a dn/dc value of 0.185 ml/g for mass calculations in SLS. Theoretical protein hydrodynamic radii were calculated from pdb files with program HYDROPRO [34].

### Protein Binding to Peptidoglycan

Protein binding to peptidoglycan was performed as described in Typas *et al.* [16]. Briefly, 10 µg of protein were incubated with a peptidoglycan suspension of *E. coli* MC1061. The peptidoglycan was pelleted, washed and resuspended in 2% SDS. The unbound fraction, the wash fraction and the resuspended pellet were analysed by SDS-18% PAGE. A control sample was realized without peptidoglycan.

### Gel Filtration

Gel filtration experiments were performed on a Superdex 200 10/300 GL and on a Superdex 75 10/300 GL for PBP3_57–577_ and PBP3_88–165_ respectively. The proteins were used at the same concentration and in the same buffer as in the crystallogenesis assay and in DLS. 200 µl of protein were injected. Standard proteins (lysozyme 14.3 kDa, trypsin inhibitor 20.1 kDa, carbonic anhydrase 31 kDa, bovine serum albumin 66.5 kDa) were used for calibration.

### Accession Numbers

The atomic coordinates for the crystal structure of PBP3_57–577_ and PBP3_88–165_ are available at the Protein Data Bank with the accession numbers PDB ID: **4BJP** and **4BJQ**.

## Results and Discussion

### Structure Determination

The crystal structure of a soluble form of PBP3, including residues 57 to 577, was solved at 2.5 Å resolution. The structure of PBP3 was solved by molecular replacement using the structure of PBP2 from *N. gonorrhoeae* (PDB id: 3equ) [35]. PBP3 crystallizes in space group P6_1_22 with one molecule in the asymmetric unit. The structure was built from residues 71 to 567 but absence of detectable electronic density did not allow structure determination for residues 93–112, 119–141, 152–162, 202–228 and 537–543. PBP3 structural information was supplemented by independently solving the Val88-Ser165 subdomain structure (see below). Final R_cryst_ and R_free_ values for the PBP3 structure determination are 19.9% and 24.5% respectively.

**Figure 1:**
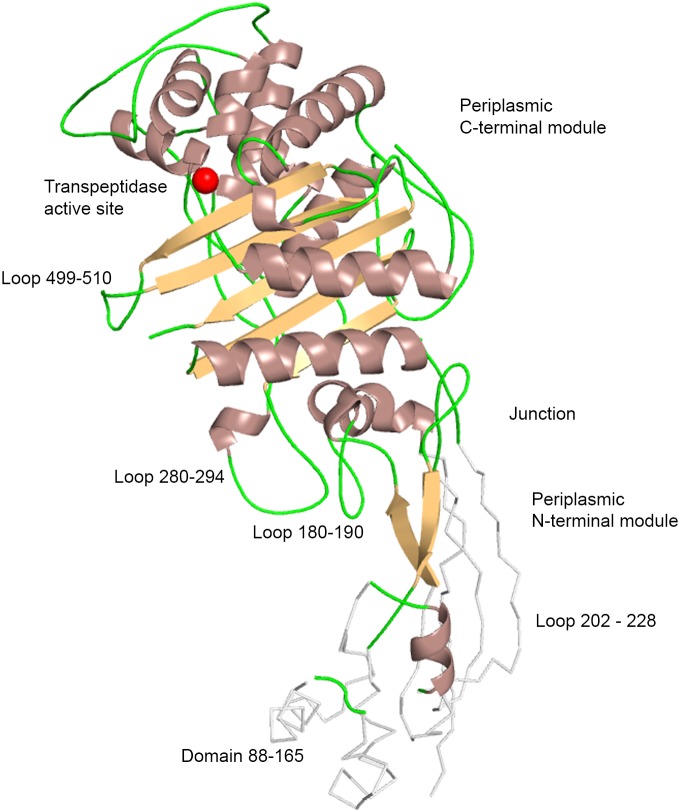
Structure of *E. coli* PBP3. Cartoon representation of the crystal structure of PBP3_57–577_. A ribbon trace of modelled loops undefined in the crystal structure is shown in grey. The active site is indicated by a red sphere. Loops discussed in the text are indicated.

The overall fold of periplasmic PBP3 is bimodular (Fig. 1). The C-terminal module is responsible for the transpeptidase activity but no clear function has been assigned yet to the N-terminal module of the construct.

### Transpeptidase Module and Active Site

**Figure 2:**
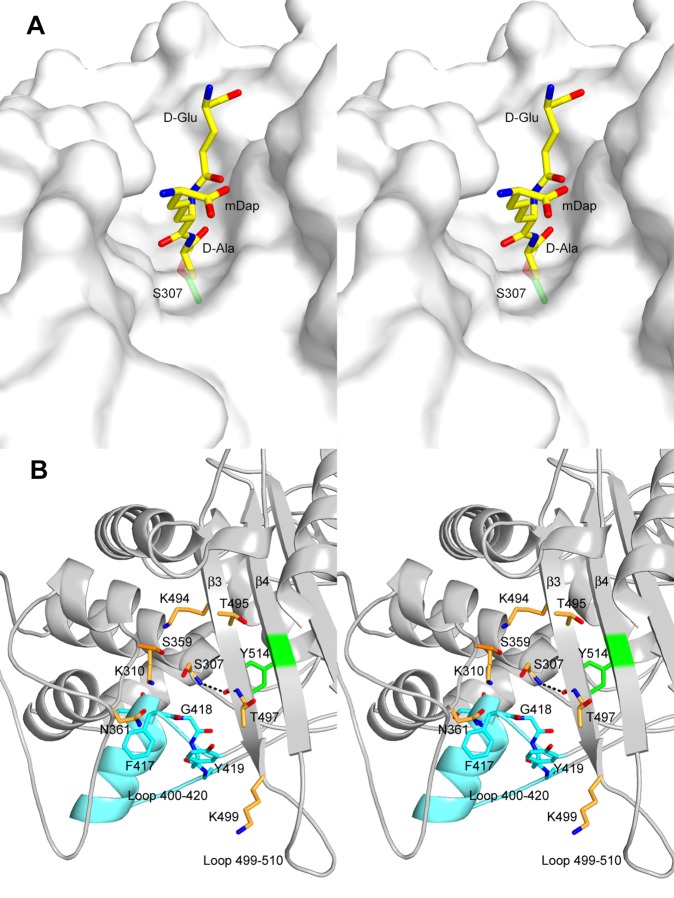
PBP3 active site. (a) Stereo view of a modelled tripeptide D-Glu-mDap-D-Ala in the active site of PBP3. The tripeptide (yellow) is modelled as an acyl-enzyme and is bonded to the active serine shown in green. (b) Stereo view of a cartoon representation of the transpeptidase active site of PBP3. The oxyanion hole is defined by the nitrogen atoms of residues 307 and 497. Loop 400–420 is shown in cyan. Nitrogen atoms are shown in blue and oxygen atoms in red.

The C-terminal module shares its overall fold with the transpeptidase domain found in all PBPs [5], [36]. Structure-based alignments of the PBP3 transpeptidase domain show little structural deviations from the corresponding domains of class B3 PBPs with r.m.s.deviations of 1.3 Å (Acitenobacter baumanii PBP3 [37]), 1.3 Å (*Pseudomonas aeruginosa* PBP3 [38] and 1.4 Å (*N. gonorrhoeae* PBP2 [35] and larger deviations for class B PBPs from other subgroups (1.7 Å, 2.1 Å, 2.1 Å, and 2.1 Å for *Mycobacterium tuberculosis* PBPA [39], *Streptococcus pneumoniae* PBP2x [40], *S. pneumoniae* PBP2b [41] and *Staphylococcus aureus* PBP2a [42], respectively). The active site responsible for the transpeptidase activity of PBP3 is located in a long groove that can accommodate the carboxy-terminal residues of the PBP3 natural substrate, the peptidoglycan stem pentapeptide L-Ala-γ-D-Glu-meso-diaminopimelic acid (mDAP)-D-Ala-D-Ala (Fig. 2a).

The transpeptidase activity of PBP3 relies on eight residues, Ser307, Lys310, Ser359, Asn361, Lys494, Thr495, Gly496 and Thr497, found with few exceptions in all penicillin-binding enzymes (Fig. 2b). These residues form three conserved sequence motifs (Ser-Xaa-Xaa-Lys, Ser-Xaa-Asn and Lys-Thr-Gly-Thr) and are also responsible for the binding of β-lactam antibiotics to the active site of PBPs [5].

The mechanism leading to linkage between the stem peptides of two glycan chains involves an acyl-enzyme formed between the active serine and the penultimate D-Ala of one stem peptide, releasing the ultimate D-Ala. In this mechanism, the nucleophylicity of the active serine Ser307 would be enhanced by Lys310, and Ser359 would be important for back-donation of the proton to the active serine during the acylation step. Deacylation involves the attack of the acyl bond by the free amine group of a second stem peptide diaminopimelic acid. Lys494 could play an important role in deacylation in concert with Ser359, as suggested for other PBPs [43]–[45].

Asn361 should be important for proper positioning of the interpeptidic amide group linking the penultimate D-Ala to the diaminopimelic acid residue. Substitution of Asn361 by a serine causes a dramatic change in pole shape [46]. The pointed polar caps observed in the *E. coli* mutant harboring this mutation appeared to be associated with the activity of PBP3. Asn361 differentiates PBP3 from its elongation homologue PBP2. The presence of an aspartic acid at this position in *E. coli* PBP2 and more generally in all PBPs of class B2 (which contains Gram negative class B PBPs associated to elongation) is a noticeable exception to the conservation of this residue in peptidoglycan synthesizing PBPs. The nature of the amino-acid should be of importance for the fine structural conformation of peptidoglycan.

Finally, both threonine residues of the Lys-Thr-Gly-Thr motif should serve as an anchor to the C-terminal carboxylate group of the pentapeptide. They are found hydrogen bonded to the penultimate D-Ala carboxylate in structures of DD-peptidases in complex with peptide fragments [45], [47].

In all ligand-free PBP-structures a water molecule is observed in the oxyanion hole. In PBP3, the oxyanion hole, defined by the amine groups of residues 307 and 497, is unexpectedly occupied by the hydroxyl group of Tyr514 that is at 2.7 Å from Ser307N and 3.25 Å from Thr497N (Fig. 2b). Sequence alignment shows that Tyr514 is unique to PBP3 among class B PBPs. The side chain of Tyr514 is free to easily rotate and liberate the oxyanion hole and should not play a particular role in transpeptidation.

The rear side of the PBP3 active site is made of residues Phe417-Gly-Tyr-Gly (Fig. 2b). The motif Tyr/Phe/Ile-Gly-Tyr/Gln-Gly and the tertiary structure of the segment 402–420 are conserved in each class of PBPs. Gly418 closes a hydrophobic pocket that can accommodate the methyl group of the penultimate D-alanine of the stem pentapeptide, conferring to PBPs a high specificity for a D-alanine as the fourth residue of the pentapeptide.

Electron density around residues 499–510, a loop that connects strands β3 and β4 close to the active site, is weak but sufficient to allow its determination. Disorder of this loop is a general property of class B PBPs whereas in other classes of PBPs, a small hairpin connects the two strands [38], [43], [47]–[50]. It could be stabilized by interactions with another protein of the divisome, e.g. for an adequate position and orientation of the active site of PBP3 along with the transpeptidase active site of PBP1b. The loop could also have a role for accompanying the displacement of the glycan chain on the surface of PBP3. In a similar manner, a disordered loop in the glycosyltransferase domain of *S. aureus* PBP2, a class A PBP homologous to PBP1b, was proposed to allow the nascent glycan chain to move processively from the donor site to the acceptor site [51].

### N-terminal Module

**Figure 3:**
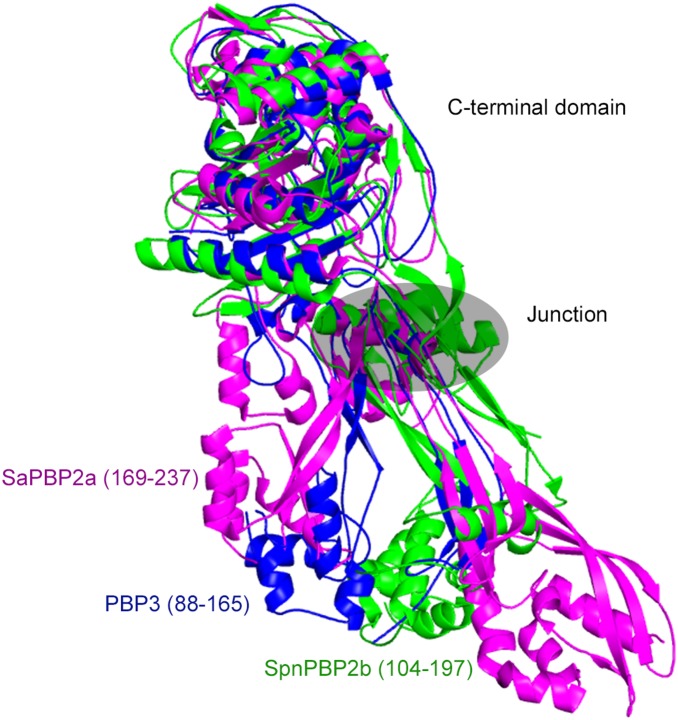
Junction between C- and N-terminal modules. Comparison between the relative orientation of N and C-terminal modules of PBP3 (blue), *S. aureus* PBP2a (magenta) and *S. pneumoniae* PBP2b (green). The C-terminal domain of SaPBP2a and SpnPBP2b are superimposed onto the C-terminal domain of PBP3. SaPBP2a (169–237) and SpnPBP2b (104–197) are equivalent to domain 88–165 of PBP3.

The N-terminal module provides three loops (180–190; 202–228; 280–294) and one subdomain (88–165) for potentially interacting with other proteins of the divisome. The residues between these loops and subdomain form a series of motifs well conserved in the primary sequence of class B PBPs [4], forming the junction between the C- and N-terminal modules and tethering the loops from the latter to the C-terminal module. Comparison with the structures of other class B PBPs shows that the relative position between the two modules can vary, suggesting that the junction between both modules is flexible. Difference between apo and acyl-enzyme structures of *P. aeruginosa* PBP3 led to the same conclusion [38]. Figure 3 shows the structures of *S. aureus* PBP2a [42] and *S. pneumoniae* PBP2b [41], with their C-terminal domain superposed onto that of PBP3, highlighting the fact that the domains equivalent to PBP3_88–165_ (domain 169–237 for SauPBP2a and domain 104–197 for SpnPBP2b) lie in different position. Class A PBPs also show a high degree of flexibility between their glycosyltansferase module and the ensemble made of the linker and the transpeptidase module [51]. Such flexibility could be necessary for the enzyme to reach its target or be required for displacement of the divisome along the septum.

The 180–190 loop forms a small β-hairpin exposing Val184 and Asp185 to the solvent. The length of this loop is characteristic of class B PBPs pertaining to the divisome (PBP3) and is much longer in class B PBPs acting during elongation (PBP2). The 280–294 loop, from the C-terminal module, is close to the 180–190 loop and is also longer in the PBPs of the elongation complex than in the PBPs of the divisome. These two loops could thus represent a specific PBP3 zone of interaction with partners of the divisome, preventing PBP3 to associate with proteins of the elongation complex or, conversely, preventing PBP2 to associate with proteins of the divisome.

Electron density is absent for segment 202–228, which again suggests that interactions with a partner protein may stabilize its tertiary structure in the divisome. Marrec-Fairley *et al.* [52] have characterized mutants of the E206-V217 segment consistent with such a role in protein interaction. R210 seems particularly important, together with residues G57, S61 and L62, for the recruitment of FtsN [53].

### PBP3_88–165_ Subdomain

Electron density was very poor for residues between Val88 and Ser185, with only some secondary structures showing up in the electron density maps. Apparent disorder of domain 88–165 is also observed in *N. gonorrhoeae* PBP2 [35] and to a lesser extent in *Pseudomonas aeruginosa* PBP3 [38], *Acinetobacter Baumanii* PBP3 [37], *Enterococcus faecium* PBP5 [54] and *S. pneumoniae* PBP2x [40], all of which are class B PBPs. Interaction of this domain with another protein of the divisome may stabilize its tertiary structure. In order to determine its three-dimensional structure, the PBP3_88–165_ domain was produced and its structure solved.

**Figure 4:**
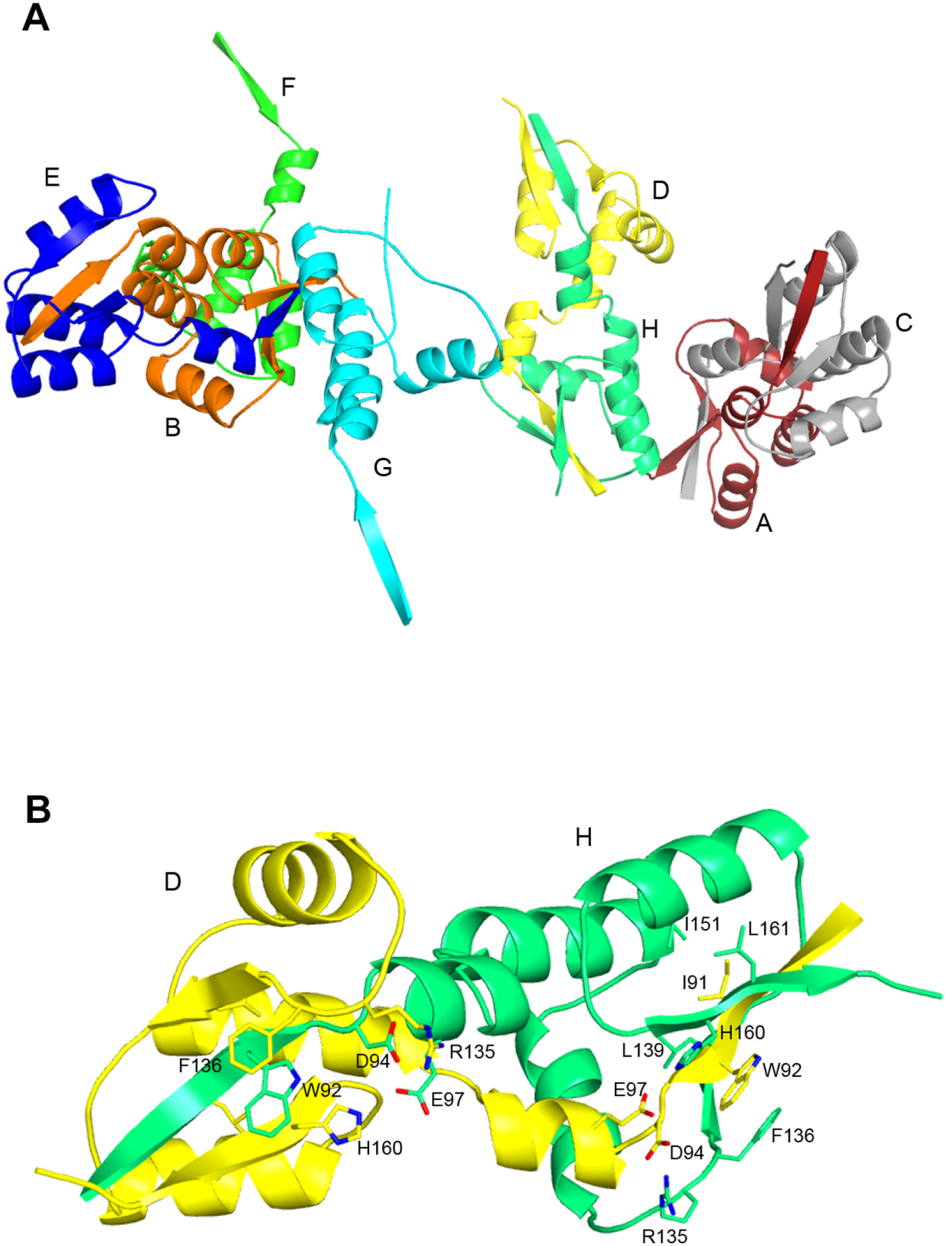
Domain swapping in PBP3_88–165_. (a) PBP3_88–165_ crystal unit cell (space group P1). The 8 chains are organized by pairs with 18 swapped residues. (b) Interactions between swapped residues from chains D (yellow) and H (green), including the hydrophobic cluster around Ile91 (Leu139, Ile151, Leu161), salt bridges (Asp94-Arg135, Glu97-His160 and an aromatic ring sandwich (His160-Trp92-Phe136). Some labels are omitted for clarity. Nitrogen atoms are shown in blue and oxygen atoms in red.

The domain crystallizes in P1 with eight molecules in the asymmetric unit. Because of the high number of copies in the asymmetric unit, molecular replacement using the closely related domain Val79-Phe151 of *P. aeruginosa* PBP3 failed to provide a solution, whatever the Molecular Replacement program used. The structure of the PBP3_88–165_ domain was eventually determined by single anomalous diffraction using a selenomethionine substituted PBP3_88–165_ crystal and refined over data collected on a crystal of the native PBP3_88–165_ protein. The electron density is well defined except for residues 132–135 in chain F and for the C-terminal residue in chains E, G and H. Final Rwork and Rfree values for the PBP3_88–165_ domain are 20.9% and 26.3% respectively. The eight molecules in the asymmetric unit are organized in four pairs with, in each pair, 18 N-terminal residues swapping into the paired molecule (Fig. 4a). The swapped residues represent a two-turn helix and a β-strand that inserts between two β-strands of the other molecule to form a three stranded β-sheet. Interactions between the two molecules are numerous and include many hydrogen bonds, salt bridges (e.g. for associated chains A and C: Asp94A-Arg135C, Glu97A-His160C), hydrophobic clusters (Ile91A is surrounded by seven leucines or isoleucines from chain C), and an aromatic ring stacking (Trp92A is sandwiched between Phe136C and His160C) (Fig. 4b). Together, residues 88–105 from one molecule and residues 106–165 from the paired molecule form a small globular domain whose tertiary structure, three anti-parallel β-strands flanked by three helices, is homologous to the equivalent domain of *P. aeruginosa* PBP3.

Mutations in the *E. hirae* PBP5_190–261_ domain, homologous to PBP3_88–165_, support the hypothesis that this domain is a good candidate to play a role in protein-protein interactions [55]. Of note is the insertion of 60 residues assembling in four helices in the corresponding domain of PBPs of subclass B5 [41], [56].

### PBP3 Dimers

PBP3 dimerization was shown *in viv*o by two-hybrid assay [19], [20] and FRET [12], and the structure of PBP3_88–157_ suggests that PBP3 dimerization could be reinforced by 3D domain swapping involving residues 88–105. The weak electronic density around domain 88–165 in the crystal of PBP3_57–577_ allows the approximate positioning of PBP3_88–165_ structure in the crystal of PBP3_57–577_. PBP3_88–165_ then faces a symmetric domain with the crystallographic axis of symmetry at the hinge point where domain swapping occurs in PBP3_88–165_, raising the possibility that swapping also occurs in the crystal of PBP3_57–577_. Domain swapping in PBP3_57–577_ would yet involve a twisting of PBP3_88–165_ domain, i.e. symmetrical PBP3_88–165_ domains would not be oriented in the PBP3_57–577_ crystal in the same manner as in the PBP3_88–165_ one.

**Figure 5:**
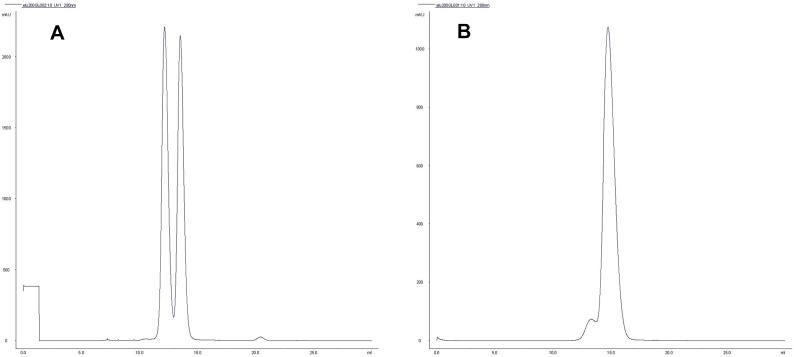
PBP3 oligomerization. (a) Chromatogram of PBP3_88–165_ gel filtration on a Superdex 75 10/300 GL. The first peak elutes at 12.16 ml and the second at 13.52 ml. Carbonic anhydrase (31 kDa) elutes at 11.05 ml and lysozyme (14 kDa) at 15.25 ml (data not shown). The buffer was 0.15 M NaCl and 0.1 M Tris, pH 8 1 mM EDTA. (b) Chromatogram of PBP3_57–577_ gel filtration on a Superdex 200 10/300 GL. The first small peak elutes at 13.3 ml, the second at 14.77 ml. Bovine serum albumin used as a standard elutes at 14.12 ml (molecular mass 67 kDa, data not shown). The masses calculated on the basis of the mass standards are 108.5 kDa for the first peak (PBP3_57–577_ dimer) and 58.5 kDa for the second peak (PBP3_57–577_ monomer). The buffer was 20 mM Tris HCl pH 8, 0.5 M NaCl.

The oligomerization state of PBP3_88–165_ and PBP3_57–577_ was investigated by Light Scattering and gel filtration. DLS and SLS experiments carried out on a solution of PBP3_88–165_ suggested a dimer in solution. The monodisperse distribution observed in DLS provided a hydrodynamic radius of 18 Å corresponding to the radius of the PBP3_88–165_ dimeric form calculated from the coordinate file whereas the average molecular mass given by SLS was 27 kDa, which is an overestimated mass of PBP3_88–165_ dimer due to the strong influence of small quantities of remaining aggregates on mass calculation. Gel filtration assays carried out with PBP3_88–165_ provided 2 peaks representing each 50% of the total protein content (Fig. 5a). The second peak represents PBP3_88–165_ dimers and the first peak accounts for higher order multimers. From these results, we conclude that, at the concentration used for crystallization, monomers of PBP3_88–165_ are absent and dimers are predominant in the solution.

DLS analysis of PBP3_57–577_ exhibited unimodal particle-size distributions with an intensity-average hydrodynamic diameter of 48 Å. Hydrodynamic radii calculated from pdb files gives 27 Å and 54 Å for a monomer or a dimer of PBP3_57–577_ respectively, suggesting that a dimer is predominant in solution. This was confirmed by SLS analysis, which provided a molecular mass of 108 kDa, corresponding to a PBP3_57–577_ dimer. In gel filtration assays, PBP3_57–577_ was mainly eluted as a monomer with 5% of dimers (Fig. 5b), which might be explained by the constant displacement of the equilibrium toward the monomer when it is separated by the size from the dimer. At the concentration used for crystallization, dimers of PBP3_57–577_ can predominate in the solution but monomers are also present and it remains unclear if PBP3_57–577_ dimerization results directly from 3D domain swapping.

3D domain swapping is frequently observed as an artefact resulting from crystallization, without bearing relevance to biological function. Because domain swapping in the PBP3_57–577_ crystal would involve a large twisting of the (88–165) domain and, also, because domain swapping should stabilize the domain and hence provide a clear electronic density in that region, the domain swapping observed in the case of PBP3_88–165_ is probably absent in the PBP3_57–577_ crystal. Moreover, in the full PBP3, domain swapping would extend from residue 105 to the amino terminus and swapping of such a large domain has never been reported. A role for domain swapping in the *in vivo* dimerization of PBP3 seems therefore elusive.

### PBP3_88–165_ Interactions

PBP1b, FtsN or FtsW are known to interact with PBP3 but a direct interaction between these proteins and the PBP3_88–165_ domain could not be detected using affinity chromatography (data not shown). Nevertheless, PBP3_88–165_ *in vitro* dimerization by domain swapping could impair the interaction, if any, of the PBP3_88–165_ domain with one of these proteins and an *in vivo* interaction of the domain with PBP1b, FtsN or FtsW cannot be discarded.

The subcomplex FtsQ/FtsL/FtsB could also be involved in the interaction with PBP3_88–165_. The N-terminal module of PBP3 appears to interact with FtsL in a two-hybrid system [20]. Lytic transglycosylases represent other potential candidates for an interaction with PBP3_88–165_. In *E. coli,* the soluble lytic transglycosylase Slt70 was shown to interact with PBP3 [57], whereas in *N. meningiditis* the membrane bound lytic transglycosylase MltA was shown to interact with PBP2Ng [58], the orthologue of *E. coli* PBP3.

**Figure 6:**
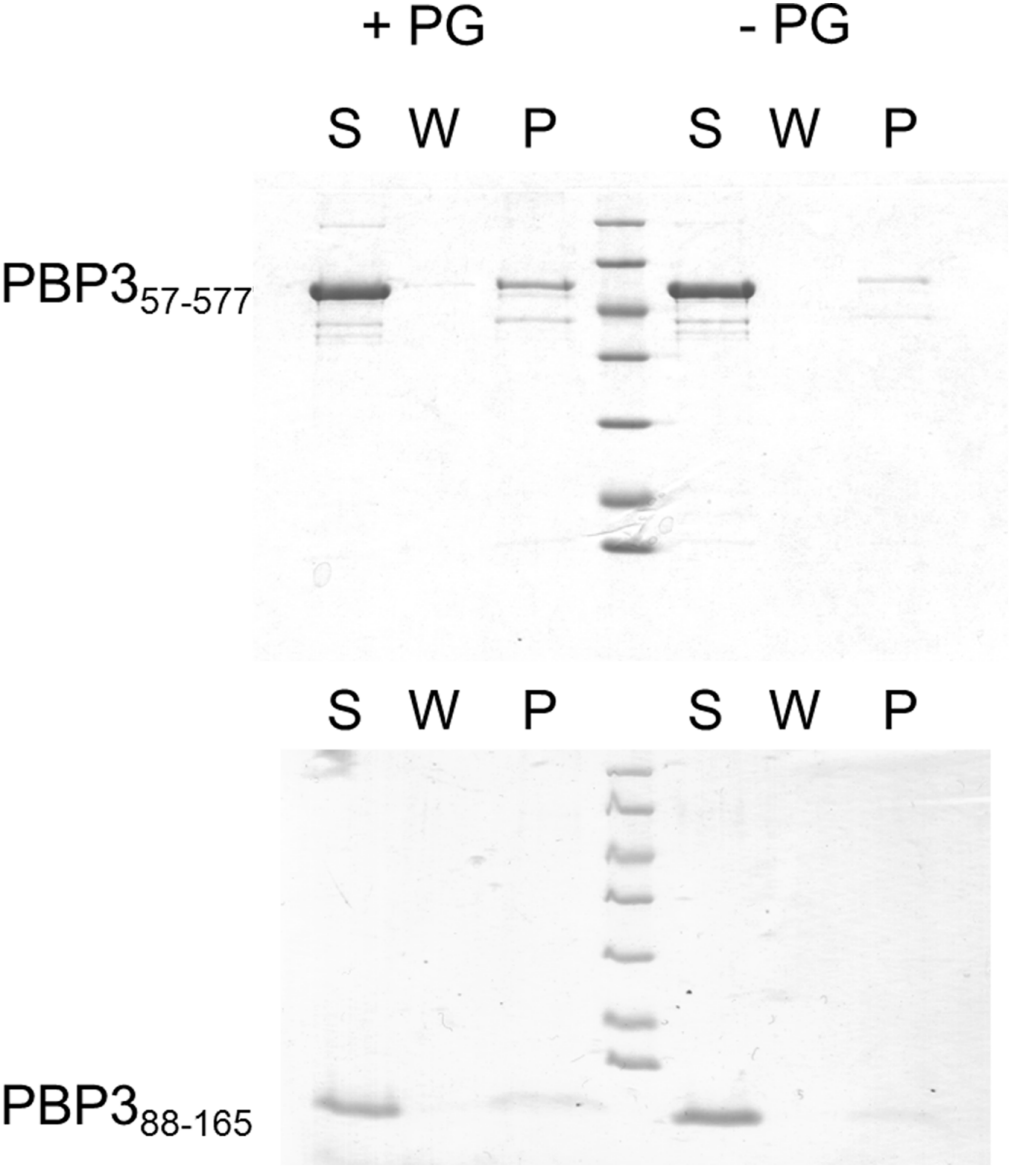
Interaction with peptidoglycan. Pulldown of PBP3_57–577_ (up) and PBP3_88–165_ (down) with and without peptidoglycan sacculi (+PG and −PG respectively). S, supernatant, W, washing step, P, pellet.

We tested the possibility that the PBP3_88–165_ domain could interact with the peptidoglycan. The binding of PBP3_57–577_ and PBP3_88–165_ to peptidoglycan sacculi was tested by a pull-down assay (Fig. 6). We showed that a part of PBP3_57–577,_ but not PBP3_88–165_, was pelleted with the sacculi indicating that it has an affinity for the peptidoglycan. On the whole, results indicate that this region of PBP3 is not essential for its interaction with the murein sacculus although PBP3_88–165_ dimerization could also perturb a possible interaction with the peptidoglycan.

## Conclusion

PBP3 interacts with many proteins and occupies a central role in the periplasmic component of the divisome. The structural information brought by the resolution of the PBP3 structure adds to the available structures of *E. coli* PBP1b, FtsQ, and FtsN carboxy terminal domain.

The modular organisation and the non-folded nature of the small loops or subdomains composing the PBP3 N-terminal module suggest that the latter could be involved in protein-protein interactions with partners of the divisome.

The structure of the PBP3_88–165_ domain, disordered in PBP3, shows a dimerization of the domain by three dimensional domain swapping that is possible but unlikely in the full length PBP3. Domain swapping in PBP3_88–165_ domain is unlikely to play a role in the *in vivo* PBP3 dimerization and a role in protein-protein interaction remains the most attractive hypothesis for this small domain.
